# Complete mitochondrial genomes of *Papilio nephelus chaon* and *Papilio epycides* (Lepidoptera: Papilionidae: Papilioninae) and phylogenetic analysis

**DOI:** 10.1080/23802359.2022.2091491

**Published:** 2022-07-04

**Authors:** Zhentian Yan, Mingjuan Lu, Site Luo, Shulin He, Wenbo Fu, Xueqian Wang, Zhenhuai Fan, Danlan Hu, Bin Chen

**Affiliations:** a Chongqing Key Laboratory of Vector Insects; Institute of Entomology and Molecular Biology, Chongqing Normal University, Chongqing, China; b School of Life Sciences, Xiamen University, Xiamen, China

**Keywords:** *Papilio nephelus chaon*, *Papilio epycides*, Papilionidae, mitochondrial genome, phylogenetic analysis

## Abstract

The complete mitochondrial genome (mitogenome) sequences of *Papilio nephelus chaon* and *Papilio epycides* were sequenced by Illumina and analyzed in this study. They are 15,287 bp and 15,012 bp in size, respectively, and contains 13 protein-coding genes (PCGs), 22 tRNA genes (tRNAs), 2 rRNA genes (rRNAs), and 1 AT-rich control region (CR). The phylogenetic relationships of 56 species in the Papilionidae were inferred based on concatenated nucleotide sequences by using Maximum Likelihood with the selected best-fit model GTR + F+R6. The phylogenetic analysis showed that *P. nephelus chaon* and *P. epycides* were located in the genus *Papilio*. This study provides a basis for further study on mitogenome and phylogenetics of the Papilionidae.

*Papilio nephelus chaon* can be found from Nepal, Sikkim, Assam to China and from Myanmar, Thailand, Cambodia to Indonesia (Zhou 1998; Wu 2001; Wu and Xu 2017; Jiang et al. 2019). *P. nephelus* wings can be used as templates to fabricate functional composite materials-metal and carbon based metal with 3-D antireflection quasi-periodicity micro structure (Cai et al. 2016). *Papilio epycides* (Hewitson 1864) is distributed in Southwest China, India, Malaysia and Indochina (Wu 2001; Wu and Xu 2017). The mitogenome data have been used to infer and analyze the phylogenetic relationships of butterflies (Qin 2017) as reliable molecular markers due to the features of maternal inheritance, stable gene composition, relative conserved gene sequence, and low recombination rate (Cameron et al. 2007; Lavrov 2007). But the complete mitogenome sequences of *P. nephelus chaon* and *P. epycides* have not been reported. In this study, we sequenced and annotated the complete mitogenomes of *P. nephelus chaon* (Genbank Number: MZ353681) and *P. epycides* (Genbank Number: MZ501807) and deduced the phylogenetics of both species and other 54 species based on mitogenomes in Papilionidae.

Adult of *P. nephelus chaon* and *P. epycides* were collected by sweep nets in Tiefo Village of Baima Town (107°20′28″E, 29°13′26″N), Wulong District, and Nanshan Botanial Park (106°63′77″E, 29°56′10″N), Nanan District in Chongqing, China. Voucher specimens were deposited in Chongqing Normal University (CQNU) (accession number 20180701001 and 20200401016, Zhentian Yan, 525201877@qq.com). The genomic DNA was extracted by using TIANamp Genomic DNA Kit (TIANGEN, Beijing, China) and the DNA library was prepared by using the Illumina Truseq™ DNA Sample Preparation Kit (Illumina, San Diego, USA) according to the manufacturer's recommendations. The prepared libraries were sequenced paired-end 150 bp on an Illumina Novaseq 6000 platform at Novogene Company (Beijing, China). NGS QC toolkit was used for quality control and to filter the low-quality reads (Patel and Jain 2012). The clean data were used to assemble the complete mitogenome by following the GetOrganelle pipeline with the ‘animal_mt’ parameter (Jin et al. 2020). Genome annotation was performed with the Mitoz annotation module (Meng et al. 2019). The annotated genomes were deposited in GenBank under with accession numbers: MZ353681 and MZ501807.

The complete mitogenomes of *P. nephelus chaon* and *P. epycides* are 15,287 bp and 15,012 in length, containing 37 typical mitogenome genes (13 protein-coding genes, 22 transfer RNAs, and 2 ribosomal RNAs genes). Overall AT content values for the mitogenomes are 80.47% and 80.07%. Both mitogenomes were assembled with high coverage (800 folded). We constructed the phylogenetics of the Papilionidae with the mitogenomes of *P. nephelus chaon* and *P. epycides* and 54 other species in Papilionidae by using maximum likelihood (ML) method implemented in IQ-TREE v2.1.2 and with the *Calingga davidis* (NC_015480) as outgroup. Each mitochondrial gene was aligned separately by the MAFFT v7.388 with default settings (Katoh and Standley 2013). The nucleotide sequences of 13 PCGs were used, and the best model GTR + F+R6 was selected by using ModelFinder (Kalyaanamoorthy et al. 2017; Minh et al. 2020).

The support values of the internal nodes in the phylogeny were inferred with 1000 bootstrapping replicates. The result shows that *P. nephelus chaon* and *P. epycides* were located in a clade including other Papilioninae species (Figure 1). This study provides important information for species identification and phylogenetic position of *Papilio* in the Papilioninae.

**Figure 1.**
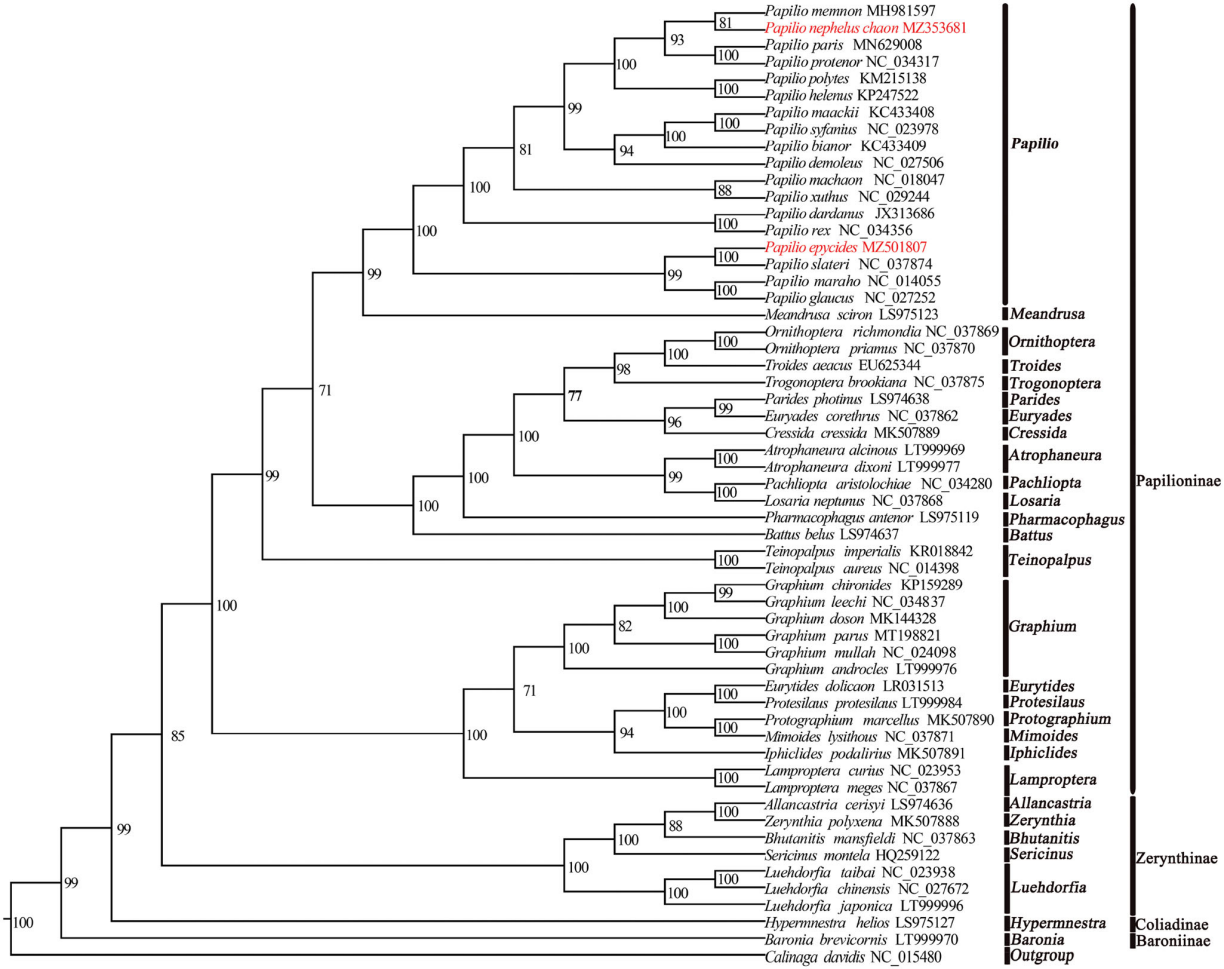
Maximum-likelihood (ML) tree based on 56 species of mitogenomes in the Papilionidae with *Calinaga davidi* as outgroup. Numbers on the nodes are bootstrap values based on 1000 replicates. The *P. nephelus* *chaon* and *P. epycides* are newly sequenced species.

## Data Availability

The genome sequence data that support the findings of this study are publicly available in GenBank of NCBI (https://www.ncbi.nlm.nih.gov/) under the accession numbers MZ353681 and MZ501807. The associated BioProject, SRA, and Bio-Sample numbers are PRJNA783429, SAMN23429721, SRR17035426 for *P. nephelus chaon* and PRJNA783428, SAMN23429720, SRR17035425 for *P. epycides*.
